# Sexually dimorphic effects of single prolonged stress on conditioned suppression behavior in Long Evans rats with no detection of altered mRNA expression of VGLUT1, GAD1, and Arc in the ventral hippocampus

**DOI:** 10.3389/fnbeh.2026.1771722

**Published:** 2026-05-28

**Authors:** Stacy R. Pitcairn, Andrew E. Tapp, Ryan M. Drenan, Jeffrey L. Weiner

**Affiliations:** Department of Translational Neuroscience, Wake Forest University School of Medicine, Winston-Salem, NC, United States

**Keywords:** conditioned suppression, RNA *in situ* hybridization, sex differences, single prolonged stress, ventral hippocampus, fear extinction

## Abstract

**Introduction:**

Women are especially vulnerable to stress-related disorders such as posttraumatic stress disorder (PTSD), yet most preclinical studies have focused on males. Rodents often exhibit sexually-dimorphic coping strategies in fear behaviors, thus complicating neurobiological interpretations. To facilitate comparisons, an operant conditioned suppression paradigm was implemented where the measure of fear is not dependent on the coping strategy. The effect of single prolonged stress (SPS) on fear behavior was assessed. Since stress has been linked to excitatory phenotypes, mRNA expression of vesicular glutamate transporter 1 (VGLUT1) and glutamate decarboxylase (GAD1) was measured to assess excitation and inhibition-related markers in the ventral hippocampus (vHip), a stress-sensitive brain region. Activity-regulated cytoskeleton associated protein (Arc/Arg3.1) was used to identify neurons activated during fear extinction. The goal of this study was to determine whether SPS produces sex differences in conditioned-suppression fear behavior and to identify accompanying molecular changes in the vHip.

**Methods:**

Thirty-two adult male and female Long Evans rats (*n* = 16/sex) were trained to lever press for sucrose on variable-interval (VI) schedules. After stable responding, a subset (8/sex) underwent single prolonged stress (SPS). All rats received fear conditioning with five presentations of a conditioned stimulus (CS)-shock pairing. Extinction sessions included baseline VI-60 responding followed by CS presentations. Fear was defined as the suppression of lever pressing during CS. Tissue containing the vHip was collected following the last session for RNA *in situ* hybridization (RNA-ISH) to examine mRNA expression of molecular markers of excitation/inhibition (VGLUT1, GAD1) and neuronal activation (Arc).

**Results:**

SPS impaired extinction in males on day 1 but had no effect in females. On day 2, males showed greater fear than females regardless of SPS. No significant effects were observed on day 3 or the recall session. No significant effects of sex or stress were observed on vHip mRNA expression of VGLUT1, GAD1, or Arc under the experimental conditions.

**Discussion:**

These findings demonstrate sex-specific effects of SPS on fear extinction measured by conditioned suppression. Future studies should explore earlier time points, additional regions (e.g., amygdala and medial prefrontal cortex) hormonal modulation, and interventions that may mitigate SPS effects in males and females.

## Introduction

Posttraumatic stress disorder (PTSD) is a complex psychiatric condition that may develop following exposure to one or more traumatic events. Unfortunately, the biological mechanisms underlying this disorder remain poorly understood and current treatments remain minimally efficacious (Krystal et al., 2017; Ressler et al., 2022). In the fifth edition of the Diagnostic and Statistical Manual of Mental Disorders, PTSD symptoms span four clusters: (1) intrusive symptoms/re-experiencing, (2) avoidance of stimuli associated with the trauma, (3) negative cognition and mood, and (4) hyperarousal/hypervigilance (American Psychiatric Association [APA], 2013). In particular, symptoms that fall into intrusive and hyperarousal clusters have been conceptualized to result from stored (i.e., non-extinguished) fear responses that can be produced in the presence of cues that resemble the stimuli present during trauma exposure (Norrholm and Jovanovic, 2018). Thus, impairments in the ability to extinguish previously conditioned fear responses have been hypothesized to underlie the maintenance of fear-related features of PTSD (Norrholm and Jovanovic, 2018; Zuj et al., 2016).

Because these symptom features are thought to reflect learned associations between environmental cues and aversive experiences, Pavlovian fear conditioning offers a well-established framework for studying these processes. In these paradigms, repeated pairings of a conditioned stimulus (CS) such as a tone, with an unconditioned stimulus (US) such as a mild footshock, elicit a conditioned fear response to the CS [reviewed in Trent et al. (2025), Wellman and Moench (2019)]. The conditioned fear response in rodents is commonly measured by freezing behavior, which is defined as the absence of all movement except for respiration (Fanselow, 1980). Extinction sessions follow, during which the CS is presented without the US and fear behavior is assessed (Trent et al., 2025; Wellman and Moench, 2019).

Single prolonged stress (SPS) is a multimodal stress paradigm that has been used as a rodent model of PTSD due to its ability to model behavioral and neurobiological symptoms associated with this disorder, such as impaired fear extinction in fear conditioning paradigms and dysregulation of the hypothalamic-pituitary-adrenal (HPA) axis (Liberzon et al., 1997; Souza et al., 2017). Importantly, the extinction impairment phenotype is highly translational and has been observed in both cued and contextual fear conditioning paradigms following stress exposure in rodents (Baran et al., 2009; Knox et al., 2012, 2018; Singewald and Holmes, 2019; Wellman and Moench, 2019) as well as in human PTSD patients (Blechert et al., 2007; Norrholm et al., 2011; Orr et al., 2000; Peri et al., 2000; Zuj et al., 2016).

Importantly, there are striking sex differences observed in PTSD that have not been consistently assessed in preclinical rodent studies. Women are twice as likely to meet PTSD criteria compared with men, even after controlling for differences in trauma type (Blanco et al., 2018; Breslau, 1997; Christiansen, 2015; Hiscox et al., 2023; Kilpatrick et al., 2013; McLean et al., 2011; Tolin and Foa, 2006). In rodent cued fear conditioning paradigms, findings regarding sex differences are very mixed, with some finding no sex differences in extinction (Kosten et al., 2006; Maes, 2002), some finding impaired extinction in females (Baker-Andresen et al., 2013; Baran et al., 2009; Fenton et al., 2014) and others finding more rapid extinction in females (Clark et al., 2019). Indeed, it has been previously argued that using freezing as the predominant measure of fear may not accurately capture other fear behaviors that may be more common in females, such as darting, which is characterized by brief, high velocity movements (Gruene et al., 2015a; Mayberry et al., 2025; Pitcairn et al., 2024; Shansky, 2018). Therefore, it has been difficult to unravel “true” sex differences in the neurocircuitry or molecular adaptations underlying fear-related behaviors due to their sexually dimorphic expression.

To facilitate comparisons between males and females, an operant conditioned suppression paradigm was implemented, as the measure of fear is not reliant on locomotor coping strategies. Conditioned suppression protocols operationally define fear as the suppression of lever pressing during the CS presentation (Kamin et al., 1963; Piantadosi et al., 2020; Piantadosi and Floresco, 2014; Sierra-Mercado et al., 2011). Thus, regardless of the locomotor coping strategy used by the rat, the same quantitative fear measure can be used to compare males and females (i.e., whether they dart or freeze, they suppress responding on the active lever). Similar to traditional fear conditioning paradigms, the conditioned suppression protocol includes a fear acquisition session, where a CS is paired with a mild footshock. The acquisition session is followed by fear extinction sessions, where the CS is presented alone. Extinction impairment can be assessed by running extinction sessions across successive days. Neuronal activation during extinction can be captured using immediate early genes such as activity-regulated cytoskeleton-associated protein (Arc/Arg3.1), which is rapidly transcribed in response to synaptic activity and has been widely used to assess neurons activated during fear learning and extinction (Maddox and Schafe, 2011; Ploski et al., 2008). Taken together, these approaches facilitate comparisons of extinction behavior and potential underlying neurobiological changes between males and females.

Given that conditioned suppression provides a comparable fear measure between males and females, the next question concerns which neural systems may underlie stress or sex-related differences. A central brain region involved in stress responsivity and fear processing is the ventral hippocampus (vHip; Kim et al., 2015; Knox et al., 2016; McHugh et al., 2004). The vHip exhibits functional connectivity with the amygdala and is implicated in both SPS-related extinction impairments (Knox et al., 2016) as well as PTSD (Abdallah et al., 2020; Ben-Zion et al., 2023; Chase et al., 2015; Ressler et al., 2022). One potential mechanism linking vHip dysfunction to maladaptive behavioral outcomes is disruption in excitatory (e.g., glutamate) and inhibitory (e.g., gamma-aminobutyric acid, GABA) neurotransmission. Stress has been shown to bias excitatory/inhibitory dynamics toward excitation/disinhibition (Elizalde et al., 2010; Santos-Silva et al., 2025). Specifically, stress has been shown to alter vesicular glutamate transporter (VGLUT1) levels and decrease levels of glutamate decarboxylase (GAD1/GAD67), a GABA synthesis enzyme, compared to unstressed controls (Banasr et al., 2017; Elizalde et al., 2010; Martisova et al., 2012; Tzanoulinou et al., 2014). Since these same transcripts have also been used to identify sex differences in excitatory and inhibitory neurotransmission (McCarthy et al., 1997), examining VGLUT1 and GAD1 mRNA provide a molecular approach to explore sex- or stress-related differences in vHip neuronal populations.

Importantly, changes in excitatory and inhibitory signaling extend beyond the molecular level and influence several behavioral domains, including fear learning and extinction (Mahan and Ressler, 2012; Steckler and Risbrough, 2012) and sleep (Meyerhoff et al., 2014) that are relevant for PTSD. Consequently, imbalances in GABA and glutamate signaling have been proposed as contributors to PTSD-related behavioral phenotypes and represent a promising avenue for novel pharmacological treatments (Lisieski et al., 2018; Steckler and Risbrough, 2012). However, it remains unclear how altered excitation/inhibition may contribute to sex- or stress-related extinction differences in conditioned suppression behavior.

Therefore, the goal of the present study was to determine whether SPS produces sex differences in conditioned-suppression fear behavior and to identify accompanying molecular changes in the vHip, including markers of excitatory/inhibitory cell types (VGLUT1, GAD1) and neuronal activation (Arc). Exposure to SPS was hypothesized to result in fear extinction impairments in both sexes when measured by conditioned suppression instead of freezing. Because Arc provides a measure of neural activation during extinction, it was also initially hypothesized that Arc expression would be elevated in glutamatergic (VGLUT1) cells, reflecting activity in these neurons among stress-exposed rats during the final extinction session. In parallel, it was hypothesized that stress exposure would cause a shift toward excitatory vs. inhibitory mRNA phenotypes in the vHip, as measured by the increased expression of VGLUT1 and/or decreased expression of GAD1.

## Materials and methods

### Subjects

A total of 32 Long Evans rats (16/sex; Envigo, Indianapolis, IN) arrived in two separate cohorts at 7 weeks old and were single-housed in standard cages (20.3 × 26.7 cm; Allentown Inc., Allentown, NJ). Male and female rats were run simultaneously and cohorts were balanced for sex and group representation. Rats were given *ad libitum* access to water and standard rodent chow (Prolab RMH 3000 Lab Diet 5P00 obtained from Lab Supply, Durham, NC). Following 1 week of facility acclimation and daily handling and weighing, rats were food restricted to 95% of their free feeding weight. Rats were weighed immediately before behavioral experiments and given pre-measured food upon completion of the sessions. Rats were maintained on a 12-h reverse light/dark cycle throughout these experiments and all behavior was assessed 1 h into the dark cycle. Cage changes occurred once weekly. All animal procedures were conducted in accordance with the Guide for the Care and Use of Laboratory Animals (National Research Council, 2011) and approved by the Wake Forest University Institutional Animal Care and Use Committee.

### Operant training

Following the acclimation period, rats began daily training in operant chambers (Med Associates, St Albans, VT). Rats were transported from their housing room to an adjacent behavioral testing room in a custom-built plastic transport carrier placed on a laboratory cart. The operant chambers were equipped with a cue light (Med Associates; ENV-221M-LED), a house light (Med Associates; ENV-215M-LED), a speaker (Med Associates; ENV-224AM-3), programmable tone generator (Med Associates; ANL-926), a pellet dispenser (Med Associates; ENV-203M-45), two levers (Med Associates; ENV-122C) and a grid floor (Med Associates; ENV-005). Rats were trained to respond on the active lever for sucrose pellets (Fisher Scientific; 45 mg Bio-Serv Dustless Precision Pellets) on a fixed ratio-1 (FR1) schedule of reinforcement for 4 consecutive days. Following this 4 day period, the inactive lever was added. A press on the inactive lever resulted in no consequence and was used to control for non-specific motor responses. Rats continued training for ∼3–4 days until they met a criterion of >80 lever presses on the active lever. Once this criterion was met, rats were trained on increasing variable-interval (VI) schedules of reinforcement (Piantadosi and Floresco, 2014). VI training began with one day of VI-15 followed by one day of VI-30. One day after VI-30 training, rats underwent a 30-min VI-60 session and continued on the VI-60 schedule for 10 days. The house light was illuminated during all training sessions.

### Single prolonged stress

Following initial training, male and female rats were randomly subdivided to undergo single prolonged stress (SPS) or the control condition. SPS was conducted in a novel testing room with overhead lights on, a fan to mask external noise, and a repeating auditory tone (1 kHz, 70 dB). Control rats were placed in a smaller room behind the testing room throughout the duration of SPS with identical environmental conditions. All rats were transported to the testing rooms in their home cages on a laboratory cart. Rats in the SPS group were first placed in hard plastic cylindrical restraints (males: ∼8” L × 3” D; females: ∼7.5” L × 2.5” D) for a duration of 2 h. Following restraint, rats were placed into a 15-min forced group swim (*n* = 4 at a time, same sex group) in a plastic tub (21 in width × 21 in length × 17 in height) filled $\frac{2}{3}$ full with 24 °C water. Following the forced swim, rats underwent a 10-min rest period on dry cloth towels. Ether exposure occurred under a fume hood to four rats at a time in a standard cage with a ventilated lid. Cotton balls with diethyl ether (Sigma-Aldrich, St. Louis, MO) were placed in the cage at a rate of 3 mL/min in a plastic barrier that prevented physical contact with the rats but allowed for vapor dissipation. Rats were removed upon loss of consciousness and lack of righting response, placed in a clean cage, monitored for recovery, and returned to their housing room for a one-week undisturbed period. Pre-measured food continued to be given daily during this period.

### Fear acquisition and extinction

Following the 1-week undisturbed period, rats underwent five VI-60 sessions to assess any effects of SPS on lever pressing before undergoing fear acquisition. During the fear conditioning session, rats were placed in the same operant boxes with no levers extended or house light illuminated. Rats were exposed to five presentations of a 30-s aversive stimulus (CS) consisting of a 9 kHz tone, flashing cue light, co-terminating with a 0.5-s footshock (0.45 mA females, 0.50 mA males) with a 3-min interstimulus interval. Shock intensities were selected based on evidence that female rats exhibit heightened sensitivity to foot shock relative to males, likely due to differences in hormonal cycling, pain circuitry, and body weight (Beatty and Fessler, 1977; Carlson and Weiner, 2023; Drury and Gold, 1978). Sex-specific intensities were therefore chosen in an attempt to equalize the subjective experience of the stressor across sexes. Twenty-four hours after the initial acquisition, rats ran in a single VI-60 session to assess any contextual effects of the operant chambers. Forty-eight hours following the initial acquisition, rats underwent a fear extinction session. Extinction sessions had the house light illuminated and both levers extended, and rats were allowed to lever press on a VI-60 schedule throughout the session. Following a 5-min VI-60 baseline came the onset of 5 CS presentations (no footshock) with a 5-min interstimulus interval. Fear behavior was operationally defined as suppression of lever pressing during CS presentations. The suppression ratio was calculated using the formula (A−B)/(A+B), where A was the number of lever presses in 30 s before the CS onset and B was the number of lever presses during the CS (Piantadosi and Floresco, 2014). A suppression ratio of 1 indicates full suppression of lever pressing during the 30-s CS and is interpreted as high fear behavior. A suppression ratio of 0 indicates no suppression during the CS. A suppression ratio of −1 can occur if rats press more during the 30-s CS than in the 30-s prior to stimulus onset. Data from operant sessions was summarized and suppression ratio was calculated per rat using a custom R package (Ortelli & Colarusso). Rats underwent three extinction sessions over three successive days. Following the 3 days of extinction sessions, daily sessions were ceased for a 48-h period. Following that period, they underwent an extinction recall session. This session is identical to prior sessions but is used to assess their fear behavior at a more remote time point, indicating the strength of the extinction memory (Milad et al., 2009).

### Tissue collection and sectioning

Because this behavioral assessment required repeated daily testing, tissue collection could only occur after the final extinction recall session. Earlier collection would have precluded assessment of extinction behavior, which was a key outcome measure. Therefore, approximately 30 min following the extinction recall session, rats were deeply anaesthetized with isofluorane before decapitation and tissue collection. Tissue was flash frozen on dry ice, embedded in tissue freezing medium (Electron Microscopy Sciences) and stored at −80 °C until sectioning. Sections containing the vHip were taken on a cryostat (Leica CM3050 S) at 12 μm and were adhered to Superfrost Plus slides.

### RNA *in situ* hybridization (RNA-ISH)

Sections were processed for RNAscope (Advanced Cell Diagnostics, ACD) multichannel RNA-ISH according to the manufacturer manual for multiplex version 2. Processed sections were mounted with ProLong Gold Antifade Mountant (ThermoFisher) and cover slipped. Probes for *Slc17a7* (Catalog No. 317001*), GAD1* (Catalog No. 316401-C2), and *Arc* (Catalog No. 540901-C3) were purchased from Advanced Cell Diagnostics.

### Image acquisition and quantification

**FIGURE 1:**
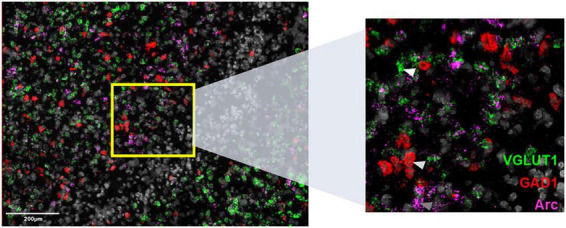
Representative RNA *in situ* hybridization (RNA-ISH) staining in the ventral hippocampus. Representative stitched image of hippocampal cells at 20 × magnification. Merged image shows DAPI nuclear stain (gray) with mRNA expression of vesicular glutamate transporter 1 (VGLUT1; green), glutamate decarboxylase 1 (GAD1; red), and activity-regulated cytoskeleton-associated protein (Arc; magenta). Arrowheads indicate example VGLUT1 (white, top arrowhead), GAD1 (light gray, middle arrowhead), and Arc (dark gray, bottom arrowhead) signal.

Slides were imaged on an Olympus IX83 inverted microscope system at 20 × magnification. All images were acquired and processed in the same manner. RNAscope fluorescent images were analyzed using QuPath (version 0.5.0). For each image, vHip regions of interest (ROIs) were manually delineated using the annotation tools in QuPath. Cell segmentation was performed using the DAPI channel to identify nuclei and define cell boundaries (Secci et al., 2023). Once satisfactory cell segmentation was achieved, RNA marker channels (FITC, TRITC, and Cy5) corresponding to VGLUT1, GAD1, and Arc were processed individually. Channel-specific thresholds were manually optimized within the ROI to ensure accurate detection of signal vs. noise across markers. For statistical analysis, raw fluorescence intensity values were exported from QuPath to allow for data preprocessing and hierarchical linear modeling using continuous signal data. A total of 12 brain slices were analyzed (*n* = 12) with 1 slice/rat (3–4 rats/condition). Representative staining is shown in Figure 1.

### Data analysis

All statistical analyses were performed in GraphPad Prism version 10.1.3 for Windows (GraphPad, Boston, MA, United States) or R (Version 4.1.3^1^) using the following packages: *dplyr* (Wickham et al., 2023), *ggplot2* (Wickham, 2016), *ggpubr* (Kassambara, 2023), *emmeans* (Lenth, 2024), *car* (Fox and Weisberg, 2019), and *lmtest* (Zeileis and Hothorn, 2002). In R, residual diagnostics (Residuals vs. Fitted, Histogram, Density, Q-Q, and Studentized Residuals plots) were assessed for each model to ensure data met statistical assumptions. These assessments revealed no substantial violations of linearity, homoscedasticity, or normality assumptions. Prior to SPS exposure, sex differences in VI-60 lever pressing were assessed using an unpaired *t*-test with Welch’s correction. The effects of sex (two levels: male vs. female) and SPS exposure (two levels: control vs. SPS) on lever pressing during the post-SPS VI-60 baseline and the VI-60 session conducted 24 h post-acquisition were assessed using two-way ANOVAs. Data for each fear extinction day were averaged across CS presentation and analyzed using a two-way ANOVA to examine the effects of sex (two levels: male vs. female) and SPS exposure (two levels: control vs. SPS) on mean suppression ratio. To examine overall extinction trajectories, suppression ratios from days 1 to 3 were analyzed using a three-way repeated measures ANOVA (sex × SPS × day). Within-session analyses were conducted using a three-way repeated measures ANOVA (sex × SPS × CS presentation) for each extinction day and the recall session. Behavioral data are expressed as the mean (M) ± the standard error of the mean (SEM). RNAscope analysis followed previously established procedures (Jin and Drenan, 2022; Yan et al., 2019). To remove rare high-intensity artifacts, a histogram-based cutoff procedure was implemented in which pixel intensities above the 99*^th^* percentile threshold were excluded from subsequent analysis. This method identifies non-biological outliers while preserving the main distribution of signal intensities. Very few images in the dataset contained such artifacts. The resulting preprocessed data was normalized as previously described (Yan et al., 2019). To assess the effects of sex and stress condition on marker expression, separate linear mixed-effects models were fit for each marker (VGLUT1, GAD1, and Arc). Because VGLUT1, GAD1, and Arc were analyzed in separate mixed-effects models reflecting distinct biological pathways, no formal correction for multiple comparisons across markers was applied. However, given that these outcomes were measured in the same animals and may be biologically related, the potential for inflated Type I error should be considered when interpreting nominal *p*-values. Each model included fixed effects for sex, stress, and their interaction, and a random intercept for subject ID to account for individual variability. The dependent variable was the normalized fluorescent intensity. This hierarchical modeling approach allows for the estimation of population-level effects while controlling for within-subject correlation.

## Results

### No effect of SPS exposure or sex on sucrose responding under a VI-60 schedule of reinforcement before nor during the post-SPS baseline

**FIGURE 2:**
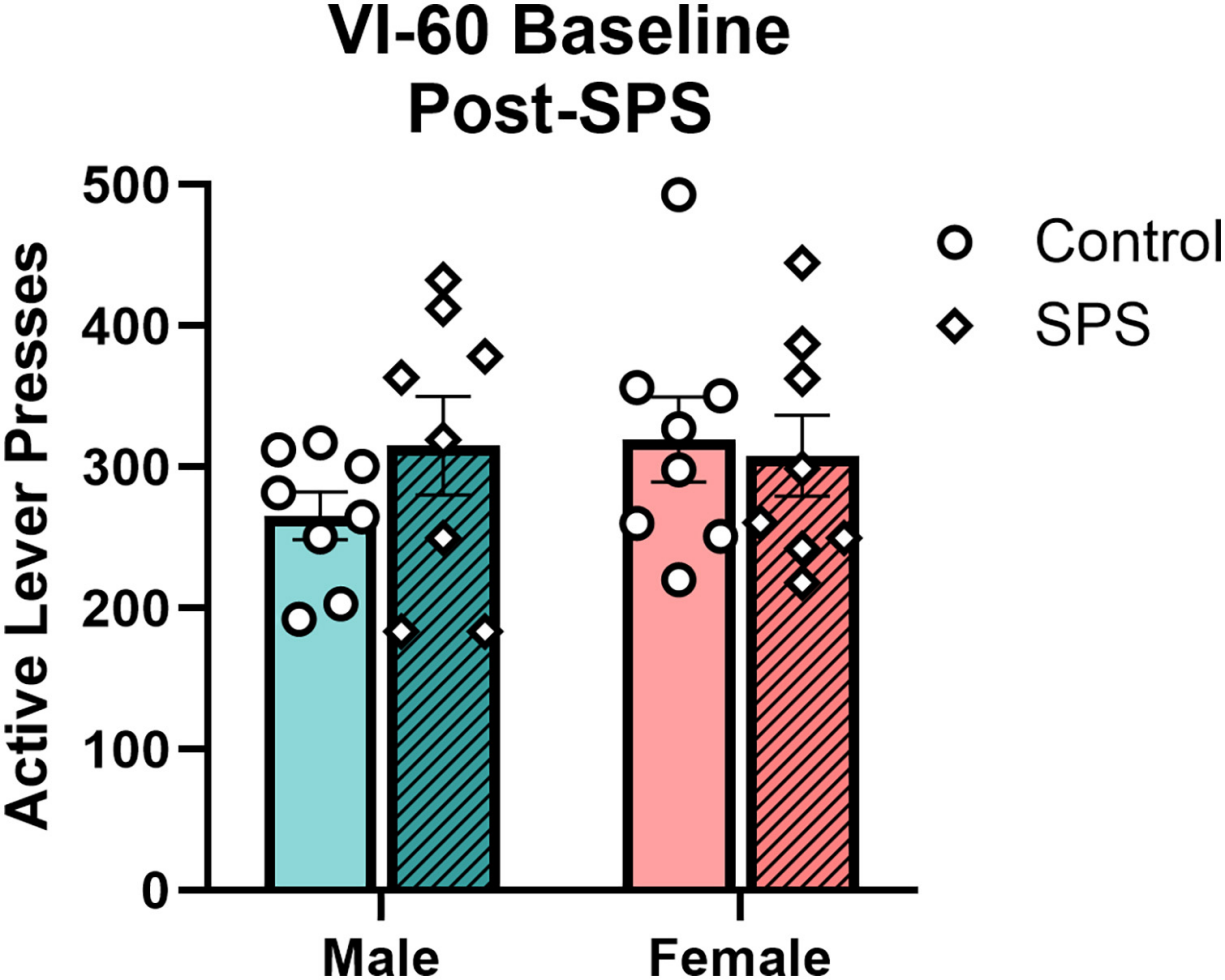
No effect of single prolonged stress (SPS) exposure or sex on mean active lever presses under a VI-60 schedule of reinforcement during a 5-day post-SPS baseline, *N* = 8/group.

Prior to SPS exposure, males and females did not differ significantly in VI-60 lever pressing [*t*(23.06) = 1.15, *p* = 0.261, data not shown], indicating comparable baseline operant responding before SPS. During the post-SPS baseline, the results revealed no significant main effects nor interactions on active lever presses. Sex did not significantly predict lever pressing [Figure 2; *F*_(1, 28)_ = 0.68, *p* = 0.416], nor did SPS exposure [*F*_(1, 28)_ = 0.46, *p* = 0.505]. The interaction between sex and SPS exposure was also not significant [*F*_(1, 28)_ = 1.17, *p* = 0.289], indicating that lever pressing in the standard VI-60 session was not affected by sex nor SPS exposure in the post-SPS baseline. To assess contextual memory for the fear acquisition environment, lever pressing was analyzed during a VI-60 session conducted 24 h post-acquisition. A two-way ANOVA (sex × SPS) revealed no significant main effect of sex [F_(1,28)_ = 1.82, *p* = 0.188], SPS [F_(1,28)_ = 2.38, *p* = 0.134], or their interaction [F_(1,28)_ = 1.93, *p* = 0.176], indicating no significant differences in responding between groups during this session (data not shown).

### SPS significantly impaired fear extinction on day 1 in males, but not females

**FIGURE 3:**
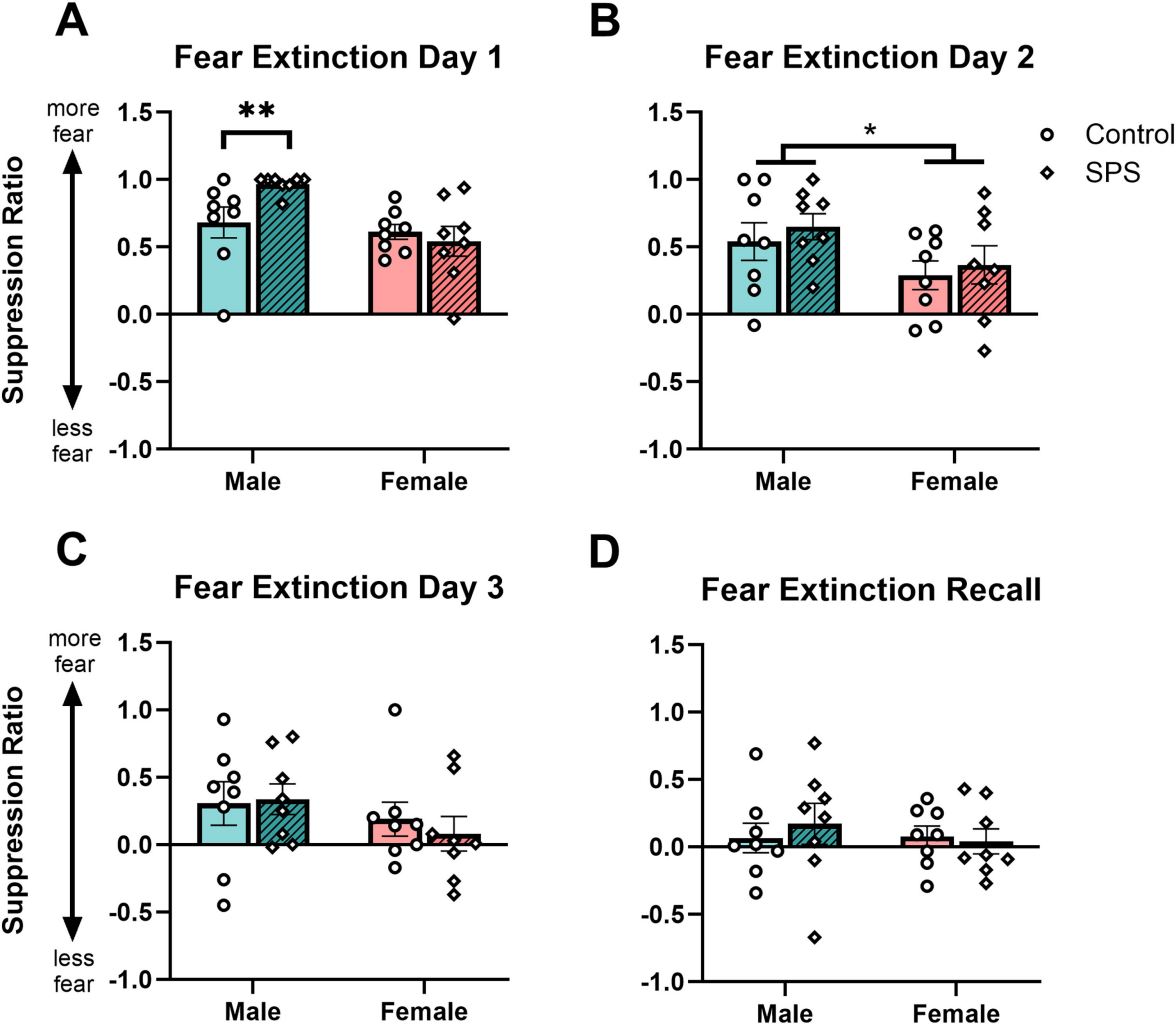
Effects of biological sex and single prolonged stress (SPS) on fear extinction measured by conditioned suppression. **(A)** Suppression ratios on day 1 of extinction. **(B)** Suppression ratio on day 2 of extinction. **(C)** Suppression ratio on day 3 of extinction. **(D)** Suppression ratio on an extinction recall session that occurred 48 h after extinction day 3. *N* = 8/group. **p < 0.01, *p < 0.05.

There was a significant main effect of sex [Figure 3A; F_(1,28)_ = 8.47, *p* = 0.007]. No main effect of SPS was observed on day 1 [F_(1,28)_ = 1.58, *p* = 0.219]. However, a significant sex × SPS interaction was observed [F_(1,28)_ = 4.40, *p* = 0.045]. To follow up on the interaction, pairwise comparisons were conducted using estimated marginal means (EMMs). Among male rats, those exposed to SPS had significantly higher suppression ratios (EMM = 0.967, SE = 0.085) than those in the control group [EMM = 0.682, SE = 0.085; *t*(28) = −2.37, *p* = 0.025]. In contrast, female rats did not show a significant difference between SPS (EMM = 0.541, SE = 0.085) and control groups (EMM = 0.613, SE = 0.085), *t*(28) = 0.59, *p* = 0.557. These results suggest that on the first day of fear extinction, SPS impaired extinction in male rats, but not in females.

### Males showed impaired extinction on day 2 compared to females, regardless of SPS

Similar to day 1, there was a significant main effect of sex [Figure 3B; F_(1,28)_ = 4.77, *p* = 0.037], indicating that regardless of SPS exposure, males had higher suppression ratios (M = 0.60, SE = 0.11) than females (M = 0.33, SE = 0.06). No significant main effect of SPS was observed [F_(1,28)_ = 0.59, *p* = 0.449] nor a sex × SPS interaction [F_(1,28)_ = 0.02, *p* = 0.895) on day 2.

### No effect of sex nor SPS exposure on fear extinction day 3 or extinction recall day

On day 3, there was no significant main effect of sex [Figure 3C; *F*_(1, 28)_ = 1.89, *p* = 0.180] nor SPS [*F*_(1,28)_ = 0.06, *p* = 0.805], nor a sex × SPS interaction [*F*_(1,28)_ = 0.06, *p* = 0.805]. This suggests that any SPS-induced impairment in males did not persist through day 3.

Similarly to day 3, there was no significant main effect of sex [Figure 3D; *F*_(1,28)_ = 0.01, *p* = 0.94] nor SPS [*F*_(1,28)_ = 0.45, *p* = 0.51], nor a sex × SPS interaction [*F*_(1,28)_ = 0.40, *p* = 0.53] during the fear extinction recall session.

**FIGURE 4:**
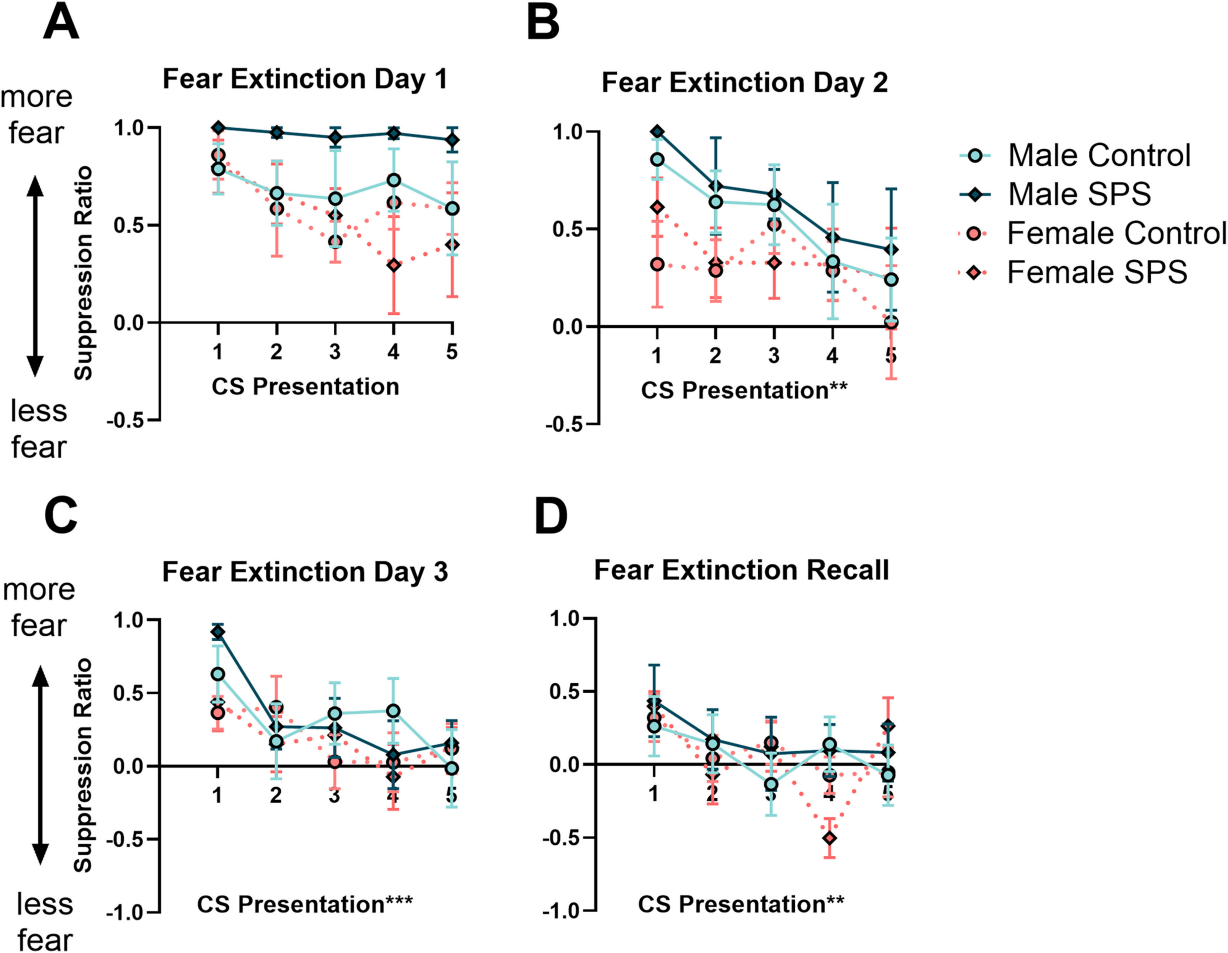
Within-session suppression ratios across conditioned stimulus (CS) presentations for each extinction day and recall session. **(A)** Day 1. **(B)** Day 2. **(C)** Day 3. **(D)** Recall. Solid lines indicate male rats and dashed lines indicate female rats. Circles represent control rats and diamonds represent SPS-exposed rats. *N* = 8/group. Error bars represent ± SEM. ***p < 0.001, **p < 0.01.

To examine whether extinction responses displayed temporal patterns within sessions, suppression ratio was analyzed across individual CS presentations using a three-way repeated measures ANOVA (sex × SPS × CS) for each day. No significant sex × CS, SPS × CS, or sex × SPS × CS interactions were observed on any extinction day or the recall session, indicating that between-group differences were consistent across CS presentations. A significant main effect of CS was observed on day 2 [Figure 4; F_(4,112)_ = 3.76, *p* = 0.007], day 3 [F_(4,112)_ = 5.71, *p* < 0.001], and the recall session [F_(4,112)_ = 3.72, *p* = 0.007], reflecting within-session reductions in suppression regardless of group. No significant main effect of CS was observed on day 1 [F_(4,112)_ = 1.65, *p* = 0.167], consistent with high and persistent fear expression early in extinction.

### Effect of sex and day across days 1–3, no group differences during recall

**FIGURE 5:**
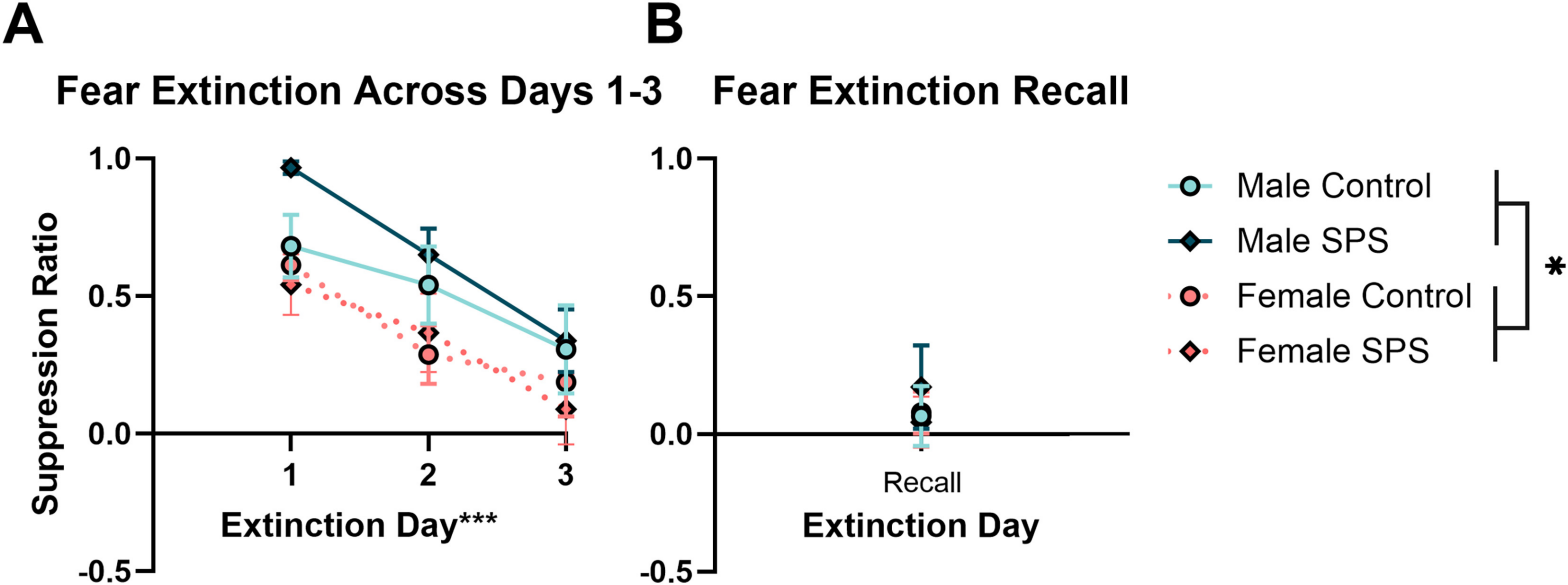
Fear extinction measured by conditioned suppression across extinction days and recall. **(A)** Mean suppression ratio across extinction days 1–3. **(B)** Mean suppression ratio during the extinction recall session conducted 48 h after extinction day 3. Solid lines indicate male rats and dashed lines indicate female rats. Circles represent control rats and diamonds represent SPS-exposed rats. *N* = 8/group. Error bars represent ± SEM. A significant main effect of sex (*p* < 0.05) and day (*p* < 0.001) were observed for days 1–3. *p < 0.05, ***p < 0.001.

To examine overall extinction trajectories, suppression ratios from days 1 to 3 were analyzed using a three-way repeated measures ANOVA (sex × SPS × day). A significant main effect of sex was observed [Figure 5A; F_(1,28)_ = 6.83, *p* = 0.014], indicating that males showed higher suppression ratios than females across extinction days. A significant main effect of day was also observed [F_(2,56)_ = 27.28, *p* < 0.001], reflecting overall reductions in suppression across sessions consistent with extinction learning. No significant main effect of SPS [F_(1,28)_ = 0.40, *p* = 0.535], nor any two- or three-way interactions involving day were observed (all *p*_*s*_ > 0.43), indicating that the pattern of extinction across days did not differ significantly by sex or SPS condition. The extinction recall session was analyzed separately using a two-way ANOVA (sex × SPS). No significant main effect of sex [F_(1,28)_ = 0.28, *p* = 0.602], SPS [F_(1,28)_ = 0.10, *p* = 0.755], or their interaction [F_(1,28)_ = 0.40, *p* = 0.534] was observed (Figure 5B).

### No significant effect of sex nor SPS exposure detected on VGLUT1, GAD1, or Arc mRNA expression

**FIGURE 6:**
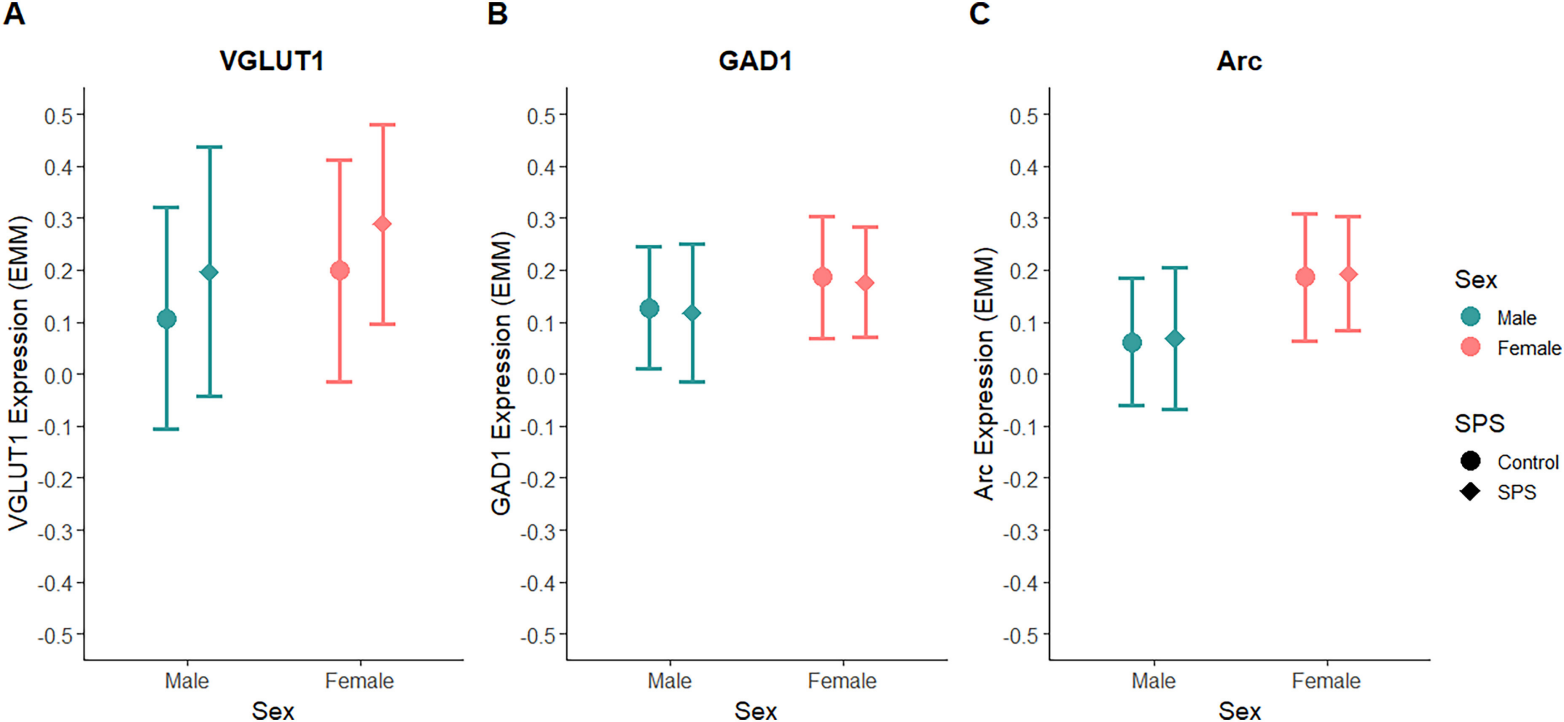
Plots showing estimated marginal mean expression of vesicular glutamate transporter 1 (VGLUT1), glutamate decarboxylase (GAD1), and activation (Arc) in the ventral hippocampus as a function of sex and single prolonged stress (SPS) condition. Visual trends in estimated marginal mean (EMM) gene expression across sex and stress conditions. **(A)** VGLUT1 expression by sex and SPS. **(B)** GAD1 expression by sex and SPS. **(C)** Arc expression by sex and SPS. A total of 12 brain slices were analyzed (*n* = 12) with 1 slice/rat 3–4 rats/condition). Color indicates sex, while symbols indicate SPS condition.

The model did not reveal any significant main effects of sex, SPS, nor their interaction on VGLUT1 expression. Since interaction between sex and SPS was non-significant, this term was removed, and the model was re-fit. In this reduced model, the effect of SPS was also not significant (β = 0.090, SE = 0.126, *p* = 0.495), nor was the main effect of sex (β = −0.092, SE = 0.128, *p* = 0.489). Estimated marginal means indicated that VGLUT1 expression was highest in females exposed to SPS (Figure 6A; *M* = 0.289, SE = 0.098, 95% CI [0.098, 0.480]) and lowest in males without SPS (Figure 6A; *M* = 0.107, SE = 0.109, 95% CI [-0.106, 0.320]). However, confidence intervals for all groups overlapped substantially, suggesting considerable uncertainty in the estimated effects.

Regarding GAD1 expression, the sex × SPS interaction term was non-significant, and the interaction term was removed. The reduced model indicated no significant main effects of sex (β = −0.059, SE = 0.071, *p* = 0.429) nor SPS (β = −0.010, SE = 0.070, *p* = 0.888). Estimated marginal means for GAD1 expression showed small differences across sex and SPS conditions. Control females had the highest mean expression (Figure 6B; *M* = 0.186, SE = 0.060, 95% CI [0.068, 0.304]), while SPS-exposed males had the lowest (Figure 6B; *M* = 0.118, SE = 0.068, 95% CI [−0.015, 0.250]). Confidence intervals for all groups again overlapped substantially.

Additionally, the effects of sex and SPS on Arc expression were examined. Again, no significant interaction was observed, so the model was re-fit with the interaction term removed. In the reduced model, the intercept was significant (β = 0.187, SE = 0.062, *p* = 0.015). Neither sex nor SPS had significant main effects (sex β = −0.125, SE = 0.073, *p* = 0.123); SPS (β = 0.007, SE = 0.072, *p* = 0.930). Estimated marginal means for Arc expression were slightly higher in females compared to males across both control and SPS conditions (Figure 6C). Control females had a mean of 0.187 (SE = 0.062, 95% CI [0.065, 0.309]), while control males had a mean of 0.062 (SE = 0.062, 95% CI [−0.060, 0.184]). Under SPS, females averaged 0.193 (SE = 0.056, 95% CI [0.084, 0.303]) and males averaged 0.069 (SE = 0.070, 95% CI [−0.069, 0.206]). Confidence intervals again overlapped considerably, reflecting uncertainty in the estimated effects. The percentage of cells that were positive for VGLUT1 and GAD1 was also quantified based on the thresholds determined in QuPath. To exclude the non-neuronal DAPI positive cells, the relative percentage of VGLUT1 was calculated by dividing count of VGLUT1+ cells by the sum of VGLUT1 and GAD counts and multiplying this value by 100. Across all rats, cells that were positive for VGLUT1 comprised an average of 65.13% while GAD1+ cells accounted for 34.87% in the ROI. Although no statistically significant main effects or interactions were detected, some consistent directional trends were observed across conditions (e.g., higher VGLUT1 expression in SPS females). Because analyses were conducted using mixed-effects models that account for cell-level clustering within animals, statistical power was necessarily reduced relative to analyses treating cells as independent observations. As a result, these null findings should be interpreted cautiously, as modest but biologically relevant effects may not have been detectable given the effective sample size.

## Discussion

The present studies sought to examine potential sex differences in fear extinction phenotypes following SPS exposure using conditioned suppression, a measure of fear behavior that does not rely on locomotor coping strategies. Sex-dependent effects of SPS were observed on fear extinction on day 1. Sex differences were observed on day 2. No effects of SPS nor sex were observed on day 3 or extinction recall. No effect of SPS nor sex was observed on lever pressing in standard VI-60 sessions following SPS.

Historically, rodent stress paradigms such as chronic unpredictable stress (Katz, 1982; Papp et al., 1991; Strekalova et al., 2004; Willner et al., 1987) and more recently SPS (Enman et al., 2015; Lee et al., 2014) have been shown to result in decreased sucrose preference and intake, a measure of anhedonia. Additionally, prior work has shown that highly stressful acute experiences produce long-lasting deficits in instrumental motivation for food in food-restricted (85%–90%) male rats when using a progressive ratio schedule of reinforcement (Derman and Lattal, 2023). Therefore, prior to any fear acquisition or extinction sessions, lever pressing for sucrose under the VI-60 schedule was assessed for any SPS-induced disruptions of active lever pressing. No effect of SPS nor sex on the number of active lever presses was observed in these sessions. This suggests that SPS does not impair general operant responding for sucrose pellets under a VI-60 schedule. However, it is important to note the schedule of reinforcement used in the present studies. VI schedules are less sensitive to motivational shifts than ratio schedules (Zuriff, 1970); therefore, the intact responding might reflect the VI schedule rather than the absence of stress effects, *per se*. One prior study showed that sleep disruption stress impairs sucrose responding in food restricted rats under a VI-30 schedule, and to a lesser extent disrupts VI-15 responding, suggesting that VI-schedule value influences the effect of stress on sucrose responding (Kirby and Kennedy, 2003). However, a VI-60 was not assessed in those experiments. Thus, future studies need to be conducted to parse out the details of how stress exposure may affect the pattern of sucrose responding under a VI-60 schedule.

On the first day of fear extinction, a sexually dimorphic effect of SPS was observed, such that SPS impaired extinction in males, but not females, in the conditioned suppression paradigm. This aligns with prior literature showing fear extinction impairments in SPS males relative to control males, but no differences between SPS and control females (Keller et al., 2015) in cued fear conditioning paradigms. However, this has not been consistently observed, as others have shown no sex differences in extinction (Kosten et al., 2006; Maes, 2002) or even impaired extinction in females (Baker-Andresen et al., 2013; Baran et al., 2009; Fenton et al., 2014). To our knowledge, the present study is the first to show this SPS-induced extinction impairment in an operant conditioned suppression paradigm that is less sensitive to sexually-dimorphic locomotor coping strategies. These findings add to the body of evidence showing that male rats may be more susceptible to extinction impairments following SPS. These results also suggest that the observed male-specific impairment is not merely an artifact of behavioral strategy differences, but may reflect neurobiological divergence.

While these sexually dimorphic effects of SPS on fear extinction may suggest an innate resilience to SPS in females, possibly due to the protective effects of estrogen (Keller et al., 2015; Peyrot et al., 2024), existing evidence supports a more nuanced framing. Within the fear domain, SPS has been shown to alter avoidance behavior during extinction, increase freezing during fear renewal, and disrupt long-term extinction retrieval in females — effects that would be missed by paradigms relying solely on freezing during extinction sessions as the primary outcome measure (Collins et al., 2023; Mancini et al., 2021; Mayberry et al., 2025). Beyond fear-related behavior, SPS produces effects in females across other PTSD-relevant domains, including acute reductions in locomotor activity (Mancini et al., 2021) and increased anxiety-like behavior on the elevated plus maze in some (Shafia et al., 2023) but not all studies (Mancini et al., 2021). At the neural level, SPS differentially alters hippocampal glucocorticoid receptor expression in females and produces extinction memory changes that are modulated by estrogen receptor signaling, demonstrating that females mount a clear neurobiological response to SPS that standard behavioral assays may not fully capture (Biddle and Knox, 2023; Keller et al., 2015). Together, these findings indicate that female responses to SPS are not absent but may be domain-specific, and underscore the importance of assessing multiple behavioral and neural outcomes to fully characterize sex-divergent consequences of traumatic stress.

Even given these examples of female sensitivity to SPS, another point worth noting is that the SPS model was originally developed and characterized in male rats (Liberzon et al., 1997). Thus, it may not be designed to fully capture stress-related vulnerabilities in females. Our lab has previously shown that female rats undergoing an early life social isolation stress paradigm require slightly modified protocols to observe the same anxiety-like behavioral phenotypes observed in the males (Ortelli et al., 2023). This raises the question of whether sex-specific adjustments to SPS parameters could enhance the model’s sensitivity for detecting stress effects in females. While the present study does not directly test this possibility, future work should explore whether modifying SPS parameters for females could reveal potential susceptibilities that may not be observable under the standard SPS protocol.

While the present findings add to evidence of male-selective vulnerability to SPS-induced extinction impairments (Keller et al., 2015), the mechanisms underlying the sex difference remain unclear. SPS has been shown to reduce excitability in the infralimbic cortex (IL) and disrupt IL inhibition of the basolateral amygdala (BLA) during extinction in males (Knox et al., 2016; Nawreen et al., 2021), alongside structural and functional changes in the BLA (Cui et al., 2008; Fang et al., 2018). Importantly, IL-BLA circuit structure predicts extinction success in males but not females, suggesting sex-specific neural mechanisms underlying fear regulation (Gruene et al., 2015b). Future studies should examine mPFC and amygdala activity in both sexes in parallel with conditioned suppression to better characterize these differences.

On day 2, only a main effect of sex was observed, suggesting that regardless of SPS, males had higher fear than females. Although SPS effects were evident in males on day 1, they were not sustained. The combined analysis of extinction days 1–3 further supports the conclusion that males showed persistently elevated fear responses relative to females throughout the extinction period, regardless of SPS exposure. This may indicate that stress may initially impair extinction, while sex-related differences persist longer due to dynamic physiological and hormonal changes. For example, it has been shown that estrogen modulates neural plasticity in brain regions critical for extinction (e.g., amygdala, hippocampus, medial prefrontal cortex) in human women as well as female rats (Zeidan et al., 2011), so it is possible that hormonal influences may contribute to sex differences in extinction even after initial effects of stress have subsided. Research on stress models, including SPS, reveals complex sex-dependent effects with distinct temporal patterns. As noted earlier, Mancini et al. (2021) found that SPS reduced locomotor activity in female and male rats initially, but 30 days later, only males showed persistent anxiety-like behaviors, enhanced startle responses, and impaired spatial memory, while females remained largely unaffected except for altered fear extinction retrieval. Similarly, Fariborzi et al. (2021) observed that prepubescent stress caused short-term synaptic changes and working memory impairments in both sexes, but only males showed persistent deficits after 30 days, while females recovered. These studies demonstrate that while initial stress responses may be similar across sexes, long-term consequences may be more male-specific. However, in the present study, the sex difference didn’t persist past extinction day 2, suggesting there may be other reasons for the observed effects. For example, one potential limitation in interpreting the day 2 sex differences is the use of slightly different shock intensities for males (0.5 mA) and females (0.45 mA) during the acquisition session. This adjustment was intentional and was based on evidence from our lab and others that female rats often exhibit heightened sensitivity to footshock (Beatty and Fessler, 1977; Carlson and Weiner, 2023; Drury and Gold, 1978; Louvart et al., 2006; but see Sneddon et al., 2023). This sensitivity is likely due to biological factors such as hormonal cycling (Drury and Gold, 1978; LaCroix-Fralish et al., 2005), differences in pain circuitry (Bartley and Fillingim, 2013; Wiesenfeld-Hallin, 2005) and body weight (Gibbs et al., 1973; Hymowitz, 1981; Marks et al., 1972). Thus, using identical shock parameters across sexes could inadvertently introduce an *a priori* sex difference. To mitigate this, the sex-specific shock intensities were chosen in an effort to equalize the subjective experience of stress/pain. Although intended to minimize the confound of biological sex differences in footshock sensitivity, this choice may nonetheless limit interpretation of the sex effect observed on day 2.

On day 3 and the recall session, no effect of SPS nor sex on fear behavior was observed. This contrasts with previous findings showing that both male and female rats exhibit extinction deficits 48 h post-acquisition (Binette et al., 2022) and contrasts with the persistent male-specific stress phenotypes reviewed in the previous section. Other contrasting work has shown that males, compared to females, responded with greater fear in extinction sessions most proximal to conditioning but subsequently showed a more rapid fear extinction over time (Clark et al., 2019). The findings from the present study also contrast with human studies showing that men exposed to a psychosocial stressor exhibited a deficit in extinction retention (48 h after acquisition) compared to men in the unstressed control group (Peyrot et al., 2024). Interestingly, in that study, the effect of stress exposure on extinction retention differed according to sex hormone status. Among women in mid-cycle and early follicular phase, exposure to stress prior to fear extinction did not impact the extinction retention pattern (Peyrot et al., 2024). However, in women using oral contraceptives, stress appears to improve the extinction retention compared to the control (Peyrot et al., 2024). These results support the consideration of sex hormone status and stress exposure during extinction learning, as both components may modulate extinction retention.

Another aspect to consider is the timing of stressor and extinction assessments. In the present study, extinction sessions occurred 2 weeks after the SPS exposure, as the 1-week period following SPS was used to conduct the post-SPS VI-60 baseline. Additionally, fear extinction day 1 occurred 48 h following the initial fear acquisition session, rather than the typical 24 h used in classic fear assays. This was because a post-acquisition VI-60 session was implemented as previously suggested (Piantadosi and Floresco, 2014). This is a longer time period than traditional fear conditioning experiments using SPS. Therefore, it is possible that the day 3 and the extinction recall observations contrast with prior literature due to the timing required of a conditioned suppression protocol.

The final aim of the present experiment sought to identify accompanying molecular changes in the vHip, including markers of excitatory/inhibitory cell types (VGLUT1, GAD1) and neuronal activation (Arc). The results of the present study revealed no significant effects of SPS or sex on VGLUT1, GAD1, or Arc under the experimental conditions. The lack of observed mRNA expression differences in VGLUT1 and GAD1 could be attributed to methodological limitations. As mentioned previously, since assessment of extinction behavior required repeated daily testing, tissue collection could only occur after the final extinction recall session. Earlier collection would have precluded assessment of extinction behavior over these days. However, by the time of tissue collection, between-group behavioral differences had resolved. Consequently, the collected tissue likely reflects neural processes associated with the culmination of extinction, rather than the time window in which SPS-induced behavioral divergence was most apparent. Therefore, the null molecular findings may be attributable to the absence of a behavioral phenotype at the tissue collection time point. Indeed, other studies have collected hippocampal tissue 24 h after cessation of fear extinction retention testing and observed molecular changes, but behavioral differences were still evident at that time point (Keller et al., 2015). However, SPS can produce relatively long-lasting molecular and circuit alterations across brain regions (Cui et al., 2008; Ding et al., 2021; Kohda et al., 2007; Nawreen et al., 2021; Serova et al., 2019; Xiao et al., 2015). Despite evidence linking behavioral measures to associated neural changes, there is also literature showing that stress-induced neural alterations are not uniformly reflected in behavior; observed effects can be specific to the circuit and behavioral paradigm used (Pennington et al., 2024; Vyas et al., 2002). Similarly, biological sex has been shown to influence hippocampal plasticity and excitation/inhibition even when behavioral findings are nuanced [reviewed in Yagi and Galea (2019)]. Although the delayed time point of tissue collection limits sensitivity to transient changes, the molecular analyses still reflect well-defined hypotheses and describe molecular features under conditions of comparable behavior. Future work should examine molecular adaptations at time points with maximal behavioral divergence to better capture transient neural signatures associated with extinction impairments.

Additionally, the effects of SPS on glutamatergic and GABAergic mechanisms are dependent on the specific brain region being examined (e.g., prefrontal cortex, amygdala, hippocampus) and the time elapsed after the stressor. For example, Fang et al. (2018) showed impaired extinction of fear memory that was associated with increased activated glutamatergic and GABAergic neurons in BLA and increased activated GABAergic neurons in central amygdala 10 days following SPS. Additionally, another study observed decreased excitatory tone in the medial PFC of SPS rats when compared to controls 3 days following SPS (Knox et al., 2010). In the same study, SPS did not alter the neurochemical profiles of either the hippocampus or amygdala (Knox et al., 2010). Taken together, these findings emphasize the importance of considering both regional specificity and temporal dynamics when evaluating the impact of SPS on mechanisms related to excitatory and inhibitory neurotransmission.

Overall, to our knowledge, these studies are the first to show SPS and sex-induced disruptions in conditioned suppression behavior, a measure of fear that is less sensitive to sexually dimorphic locomotor coping strategies than conditioned freezing. These findings also add to the body of evidence showing that male rats are more susceptible to extinction impairments following SPS. Although the shock-intensity difference remains a possible contributing factor, the results suggest that the observed male-specific impairment is not merely an artifact of behavioral strategy differences. These findings emphasize the importance of studying sex as a biological variable and behavioral measures beyond conditioned freezing. The male-specific SPS impairment was not sustained, as only a sex difference was observed on the second extinction day, and no effects of SPS or sex were detected by the third extinction session or during extinction recall. At the molecular level, no SPS- or sex-dependent changes were detected in vHip expression of VGLUT1, GAD1, or Arc at the time of tissue collection, underscoring the necessity of aligning molecular measurements with the time point in which behavioral effects are most pronounced. Future studies should explore earlier time points, additional brain regions (e.g., amygdala and medial prefrontal cortex), hormonal modulation, and interventions that may mitigate SPS effects.

## Data Availability

The raw data supporting the conclusions of this article will be made available by the authors, without undue reservation.

## Footnotes

1
http://www.r-project.org/
